# Biological investigation of *N*-methyl thiosemicarbazones as antimicrobial agents and bacterial carbonic anhydrases inhibitors

**DOI:** 10.1080/14756366.2022.2055009

**Published:** 2022-03-24

**Authors:** Ilaria D’Agostino, Githa Elizabeth Mathew, Paola Angelini, Roberto Venanzoni, Giancarlo Angeles Flores, Andrea Angeli, Simone Carradori, Beatrice Marinacci, Luigi Menghini, Mohamed A. Abdelgawad, Mohammed M. Ghoneim, Bijo Mathew, Claudiu T. Supuran

**Affiliations:** a Department of Pharmacy, “G. d’Annunzio” University of Chieti-Pescara, Chieti, Italy; b Department of Pharmacology, Grace College of Pharmacy, Palakkad, India; c Department of Chemistry, Biology and Biotechnology, University of Perugia, Perugia, Italy; d Neurofarba Department, University of Florence, Sesto Fiorentino, Italy; e Department of Pharmaceutical Chemistry, College of Pharmacy, Jouf University, Sakaka, Saudi Arabia; f Department of Pharmacy Practice, Faculty of Pharmacy, AlMaarefa University, Ad Diriyah, Saudi Arabia; g Department of Pharmaceutical Chemistry, Amrita School of Pharmacy, Amrita Vishwa Vidyapeetham, AIMS Health Sciences Campus, Kochi, India

**Keywords:** Thiosemicarbazones, antimicrobial agents, *Escherichia coli*, dermatophytes, carbonic anhydrases

## Abstract

The enormous burden of the COVID-19 pandemic in economic and healthcare terms has cast a shadow on the serious threat of antimicrobial resistance, increasing the inappropriate use of antibiotics and shifting the focus of drug discovery programmes from antibacterial and antifungal fields. Thus, there is a pressing need for new antimicrobials involving innovative modes of action (MoAs) to avoid cross-resistance rise. Thiosemicarbazones (TSCs) stand out due to their easy preparation and polypharmacological application, also in infectious diseases. Recently, we reported a small library of TSCs (**1–9**) that emerged for their non-cytotoxic behaviour. Inspired by their multifaceted activity, we investigated the antibacterial, antifungal, and antidermatophytal profiles of derivatives **1–9**, highlighting a new promising research line. Furthermore, the ability of these compounds to inhibit selected microbial and human carbonic anhydrases (CAs) was assessed, revealing their possible involvement in the MoA and a good selectivity index for some derivatives.

## Graphical Abstract

**Figure:**
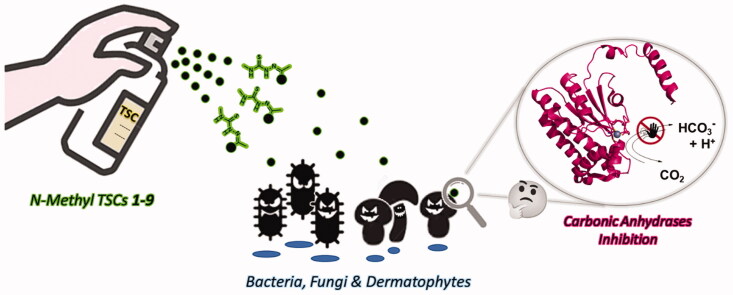

## 1. Introduction

Antimicrobial resistance (AMR) has been defined as “a slow tsunami” able to fast blow all currently available antibiotic treatments^1^. The recent public health emergency of the COVID-19 pandemic contributed to the dramatic increase of the AMR phenomena^2^ due to the high rate of prescribed antibiotics in hospitalised patients, despite the causative agents being identified in less than one-third of the cases^3^. In addition, COVID-19 containment campaigns led to an overuse of sanitisers and biocides, promoting cross-resistance and reduction or loss of antibiotic sensitivity^4^^,^^5^. Several cases of secondary infections from the bacteria *Pseudomonas aeruginosa* and *Staphylococcus aureus*^6^^,^^7^ and the opportunistic fungi from *Candida* species^8^^,^^9^ were recorded and are alerting the scientific community. Moreover, besides systemic fungal infections, mycoses of skin, nails, and hair caused by dermatophytes are generating a great concern, since they are estimated to affect a large percentage of the global population^10^. Indeed, even if not lethal, these infections negatively impact the quality of life of patients and can become invasive in immunocompromised and predisposing conditions^11^. However, although the link between inflammatory skin conditions and COVID-19 is not proven, patients with a defective skin barrier are more susceptible to other infections, worsening the risk of contracting COVID-19-related diseases^12^. Furthermore, resistant phenotypes along with non-standardized treatment protocols impair the outcomes^11^, especially for diseases due to *Trichophyton* species, one of the commonest dermatophytes infecting mammals.

In the last two years, no new antibiotics received the Food and Drug Administration (FDA) approval^13^ and few Research & Development (R&D) projects focussed on new therapeutic strategies against the microbial infections referred as to “high-risk” by the World Health Organisation (WHO) and the Centre of Disease Control (CDC) ^14^^,^^15^. Moreover, the Antimicrobial Resistance Benchmark of 2021 agrees in noticing that the number of new antimicrobials developed is far minor than those losing their effectiveness and few or no drug candidates against *Candida* species and *H. pylori*, respectively, are in advanced clinical trials to date^15^.

In this frame, drug discovery efforts are addressed on the search for new antimicrobial agents endowed with chemical and mechanistic innovation to enlarge the smaller and smaller clinically available drugs armamentarium and tackle AMR. Among the most promising pharmacological targets, the spotlight has turned to the carbonic anhydrases (CAs), ubiquitary metalloenzymes involved in the CO_2_/HCO_3_^–^ balance in multiple biological pathways, since their high conservation rate and druggability^16–18^.

Also in bacterial and fungal kingdoms, CAs play crucial roles in the growth, pathogenicity, and virulence, and structural differences of the four microbial CAs classes (α, β, γ, and ι) with respect to α-isoforms in humans (hCA I and hCA II) were observed, laying the foundation for the development of highly selective inhibitors^17–23^. Notably, relevant proof-of-concept between the CA inhibition and the *in vivo* antibacterial efficacy confirmed this valuable strategy^24–27^ and several CAs from *S. aureus, E. coli*, *P. aeruginosa, H. pylori,* and other microorganisms were recently detected and characterised, allowing the design of nanomolar inhibitors^16^^,^^20^^,^^28–34^.

Thiosemicarbazones (TSCs) are a class of compounds widely explored in the medicinal chemistry field^35^ due to their relevant broad-spectrum biological activity, often related to their ability to complex metals^36^^,^^37^, such as enzyme cofactors and essential elements for cell life. Besides other biological activities, notable antimicrobial effects against both bacterial^38–42^ and fungal^43–45^ species were reported. Moreover, the high chemical versatility as key intermediates for heterocycles preparation, along with their fast and straightforward syntheses^46^ allowed to expand the TSC class and further explore their pharmacological potential.

**Figure 1.**
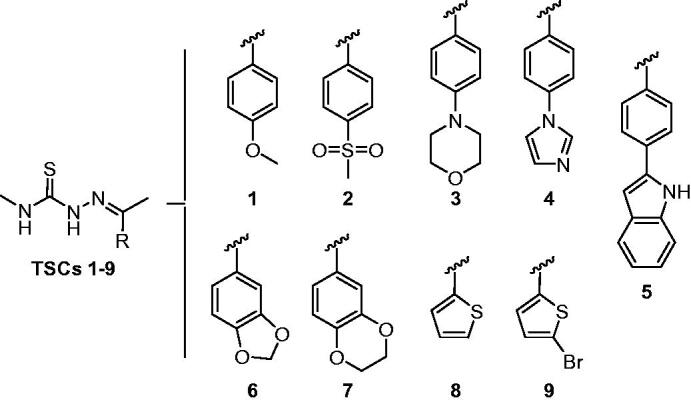
Molecular structures of *N*-methyl TSCs **1–9** investigated in this work.

Recently, focussed libraries of *N*-methyl TSC derivatives have been investigated as anti-MAO-B agents for a perspective application in Parkinson’s disease treatment, resulting in a relevant micromolar inhibitory activity^47^^,^^48^. In particular, compounds **1**–**9**, depicted in Figure 1, emerged for their non-toxic profiles, opening the door to different scenarios of biological investigation.

Hence, TSCs **1**–**9** were tested as antimicrobial agents towards Gram-negative and Gram-positive bacterial strains and fungal microorganisms, including representatives of opportunistic *Candida* species and dermatophytes. In the end, the ability of this library to inhibit carbonic anhydrases was assessed through the well-validated stopped-flow CO_2_ hydrase assay. The expectation of interaction with these enzymes is justified by the propensity of TSC function to act as ligand donors, coordinating different transition metal ions, such as zinc or others^49–51^, by its hydrazine nitrogens and sulphur atom. Furthermore, the presence of an additional heteroatom close to the TSC function (as the thiophene sulphur in compounds **8** and **9**) is hypothesised to confer an increased flexible multi-dentate chelating ability, generating complexes with different coordination geometries^52^^,^^53^.

## 2. Experimental

### 2.1. Chemistry

Compounds **1–9** were prepared *via* a clean and high-yielding synthetic approach by reacting the suitable methylketones and *N*-methylthiosemicarbazide in ethanol with a catalytic amount of acetic acid as previously reported^48^.

### 2.2. Antimicrobial susceptibility testing

*In vitro* antimicrobial activity of TSCs **1**–**9** was assessed against 8 Gram-negative and Gram-positive bacterial strains, namely *B. subtilis* (PeruMycA 6), *S. aureus* (ATCC 6538), *B. cereus* (ATCC 12826), *E. coli* (ATCC 10536), *E. coli* (PeruMycA 2), *E. coli* (PeruMycA 3), *P. aeruginosa* (ATCC 15442), and *S. typhi* (PeruMycA 7); 4 yeasts from *Candida* species, namely *C. tropicalis* (YEPGA 6184), *C. albicans* (YEPGA 6379), *C. albicans* (YEPG 6138), and *C. parapsilosis* (YEPGA 6551); and 10 dermatophyte species, namely *T. mentagrophytes* (CCF 4823), *T. tonsurans* (CCF 4834), *T. rubrum* (CCF 4879), *T. rubrum* (CCF 4933), *T. mentagrophytes* (CCF 5930), *A. quadrifidum* (CCF 5792), *A. crocatum* (CCF 5300), *A. curreyi* (CCF 5207), *A. insigulare* (CCF 5417), and *A. gypseum* (CCF 6261).

The tested microbial strains are from the ATCC (from https://www.atcc.org/), CCF (Culture Collection of Fungi, from the Department of Botany, Faculty of Science, Charles University in Prague, Prague, Czech Republic), and PeruMycA (from the Department of Chemistry, Biology and Biotechnology, University of Perugia, Italy) cultures and are available upon request. The antimicrobial activities of TSCs **1–9** were compared to reference drugs: ciprofloxacin (CIP), fluconazole (FLU), and griseofulvin (GRI) for antibacterial, antifungal, and antidermatophytal activities, respectively. Tested compounds were prepared as 3 mg/mL stock solution in dimethylsulphoxide (DMSO) and then used in the range 1.56–50 µg/mL. Each experiment of Minimum Inhibitory Concentration (MIC) evaluation was performed in triplicate. Geometric means and MIC ranges were calculated. MICs on bacterial strains were determined according to the broth microdilution method of the Clinical and Laboratory Standards Institute (CLSI) ^54^. Susceptibility testing against yeasts and filamentous fungi was performed according to the CLSI protocols^55–57^. The experimental conditions were already reported^58–60^.

### 2.3. *In silico* targets investigation

Protein targets prediction was performed for representative compounds of the TSC library through the online PPB2 tool (https://ppb2.gdb.tools, accessed on 25 January 2022) by inserting compounds smiles using compound-protein targets associations in the ChEMBL22 database along with the ECfp4 Naïve Bayes Machine Learning model produced on the fly with 2000 nearest neighbours from the Extended Connectivity fingerprint ECfp4 (NN(ECfp4)+NB(ECfp4)) and the Shape and Pharmacophore fingerprint Xfp NN(Xfp)+NB(ECfp4), following to developers instructions.

### 2.4. Expression and purification of the bacterial CAs

CAs of interest were produced in *E. coli* (DE3) competent cells through the suitable vector expression and, then, purified, as previously reported^61^.

### 2.5. Carbonic anhydrase inhibition studies

An Applied Photophysics stopped-flow instrument was used for assaying the CA catalysed CO_2_ hydration activity. Phenol red (at a concentration of 0.2 mM) was used as indicator, working at the absorbance maximum of 557 nm, with 20 mM 4-(2-hydroxyethyl)-1-piperazineethanesulfonic acid (HEPES) (pH 7.40) for α-CA class as buffer, 20 mM tris(hydroxymethyl)aminomethane (TRIS) (pH 8.30) for β- and γ-CA classes as buffer, and 20 mM Na_2_SO_4_ to maintain constant the ionic strength, following the initial rates of the CA-catalysed CO_2_ hydration reaction for a period of 10–100 s. The CO_2_ concentrations ranged from 1.7 to 17 mM for the determination of the kinetic parameters and inhibition constants. The uncatalyzed CO_2_ hydration was not subtracted from these curves and accounts for the remaining observed activity even at a high concentration of inhibitor, being in the range of 16–25%. However, the background activity from the uncatalyzed reaction is always subtracted when IC_50_ values are obtained by using the data analysis software for the stopped-flow instrument. Enzyme concentrations ranged between 5 and 12 nM. For each inhibitor, at least six traces of the initial 5–10% of the reaction were used for determining the initial velocity. The uncatalyzed rates were determined in the same manner and subtracted from the total observed rates. Stock solutions of the inhibitor (0.1 mM) were prepared in distilled-deionized water and dilutions up to 0.01 nM were done thereafter with the assay buffer. Inhibitor and enzyme solutions were preincubated together for 15 min at room temperature before the assay, to allow for the formation of the enzyme-inhibitor complex. The inhibition constants (*K*_I_s) were obtained by non-linear least-squares methods using PRISM 3 and the Cheng-Prusoff equation as reported earlier and represent the mean from at least three different determinations. All CA isoforms were recombinant proteins obtained *in house*, as already reported^28^^,^^31^^,^^33^^,^^62–64^.

### 2.6. *In silico* absorption evaluation

The blood-brain barrier (BBB) permeation properties of TSCs **1–9** were evaluated using the online BBB prediction online tool (https://www.cbligand.org/BBB, accessed on 25 January 2022) and the scores were determined using the SVM (support vector machine) algorithm along with the MAACS fingerprint. TSCs **2–9** resulted in having “positive” BBB permeation properties, as shown by the obtained score values > 0.02, whereas compound **1** was found “negative”, but “positive” by all the other available algorithms. The SwissADME server (http://www.swissadme.ch/index.php, accessed on 29 January 2022) was used to predict the Log *K*_p_ values for skin permeation assessment. All tested compounds displayed values ranging from −6.05 to −7.29 cm/sec, resulting in a good permeative property.

## 3. Results and discussion

### 3.1. Antimicrobial activity of TSCs 1–9 against bacterial, fungal, and dermatophyte species

The polypharmacology of TSCs prompted us to evaluate the antimicrobial profiles of derivatives **1**–**9** against a panel of microorganisms (*in cellulo*) and carbonic anhydrases (*in vitro*).

In particular, the antibacterial activity of **1**–**9** was assessed by determining MIC values on three Gram-positive strains (*B. subtilis*, *S. aureus*, and *B. cereus*) and five Gram-negative (three *E. coli* strains, *P. aeruginosa*, and *S. typhi*), including environmental isolates collected in Perugia (Italy) and ciprofloxacin (CIP) was used as a reference control. Data are reported in Table 1.

**Table 1.** MICs of TSCs **1**–**9** on representative Gram-positive and Gram-negative bacterial strains, including environmental isolates.

Cpd	MIC (µg/mL)*							
	Bsu^a^	Sau	Bce	Eco	Eco 1^b^	Eco 2^b^	Pae	Sty^a^
**1**	>50	39.68 (25–50)	>50	9.92 (6.25–12.5)	9.92 (6.25–12.5)	>50	>50	>50
**2**	>50	>50	>50	4.95 (3.12–6.25)	7.87 (6.25–12.5)	>50	>50	>50
**3**	>50	>50	>50	7.87 (6.25–12.5)	7.87 (6.25–12.5)	>50	>50	>50
**4**	>50	39.68 (25–50)	>50	19.84 (12.5–25)	19.84 (12.5–25)	>50	39.68 (25–50)	>50
**5**	>50	>50	>50	2.45 (1.52–3.12)	7.87 (6.25–12.5)	>50	39.68 (25–50)	>50
**6**	>50	>50	>50	15.75 (12.5–25)	39.68 (25–50)	>50	>50	>50
**7**	>50	39.68 (25–50)	>50	4.95 (3.12–6.25)	7.87 (6.25–12.5)	>50	>50	>50
**8**	>50	39.68 (25–50)	>50	9.92 (6.25–12.5)	7.87 (6.25–12.5)	>50	39.68 (25–50)	>50
**9**	>50	>50	>50	31.49 (25–50)	39.68 (25–50)	>50	>50	>50
CIP	<0.125	0.62 (0.49–0.98)	<0.125	<0.125	1.23 (0.98–1.95)	0.62 (0.49–0.98)	1.23 (0.98–1.95)	0.38 (0.24–0.49)

*MICs are expressed as the geometric mean of three independent replicates. MIC range concentrations are reported within brackets. Tested strains are: Bsu: *B. subtilis* PeruMycA 6, Sau: *S. aureus* ATCC 6538, Bce: *B. cereus* ATCC 12826, Eco: *E. coli* ATCC 10536, Eco 1: *E. coli* PeruMycA 2, Eco 2: *E. coli* PeruMycA 3, Pae: *P. aeruginosa* ATCC 15442, and Sty: *S. typhi* PeruMycA 7. ^a^Clinical isolates, ^b^Environmental isolates. Ciprofloxacin (CIP) was used as a reference control in these assays.

All the compounds resulted to be inactive at the highest tested concentration tested (50 µg/mL) versus *B. cereus* and *B. subtilis*, *E. coli*, and *S. typhi* isolates. Interestingly, a different activity profile is noticed among the 3 different strains of *E. coli*: TSCs **1–8** displayed potent activity against *E. coli* ATCC 10536 and PeruMycA 2 (MICs ranging from 2.45 to 19.84 µg/mL), meanwhile, a second isolate, PeruMycA 3, resulted to be not susceptible to the tested compounds, suggesting that the latter could have developed resistant phenotypes to TSCs and a specific molecular target could be involved in the mechanism of action. Otherwise, observing data in Table 1, a different trend of antibacterial activity on *S. aureus* and *P. aeruginosa*: only compounds **4** and **8**, endowed with an imidazole or a thiophene ring, respectively, were found to exert a moderate activity with a MIC value of 39.68 µg/mL on both the strains.

STCs **1**–**9** were also tested against yeast clinical isolates: two strains belonging to *C. albicans* (YEPGA 6379 and YEPGA 6183), *C. tropicalis* (YEPGA 6184), and *C. parapsilosis* (YEPGA 6551) with respect to fluconazole as a reference drug (data not shown). All compounds resulted to be inactive at the highest tested concentration (MIC values >50 µg/mL), with the exception of compound **8** which showed a notable MIC value of 9.92 (as the geometric mean of an experiment in triplicate).

Additionally, compounds **1**–**9** were tested against multiple species of dermatophytes (*Trichophyton* spp. and *Arthroderma* spp.) and MIC values are reported in Table 2 with respect to griseofulvin (GRI) as a reference drug.

**Table 2.** MICs of TSCs **1**–**9** on representative dermatophytal strains.

Cpd	MIC (µg/mL)*									
	Tmen	Tto	Trub	Trub	Tmen	Aqu	Acro	Acu	Ains	Agyp
**1**	>50	9.92 (6.25–12.5)	>50	19.84 (12.5–25)	>50	2.45 (1.56–3.12)	4.95 (3.12–6.25)	2.47 (1.56–3.12)	9.92 (6.25–12.5)	19.84 (12.5–25)
**2**	>50	>50	>50	39.68 (25–50)	>50	4.95 (3.12–6.25)	2.47 (1.52–3.12)	<1.56	1.96 (1.56–3.12)	2.47 (1.52–3.12)
**3**	>50	2.45 (1.56–3.12)	>50	39.68 (25–50)	>50	>50	2.47 (1.52–3.12)	2.47 (1.56–3.12)	39.68 (25–50)	39.68 (25–50)
**4**	18.84 (12.5–25)	7.87 (6.25–2.5)	>50	9.92 (6.25–12.5)	18.84 (12.5–25)	4.95 (3.12–6.25)	2.47 (1.52–3.12)	4.95 (3.12–6.25)	2.47 (1.56–3.12)	39.68 (25–50)
**5**	>50	2.45 (1.56–3.12)	>50	19.84 (12.5–25)	9.92 (6.25–12.5)	2.47 (1.56–3.12)	2.47 (1.52–3.12)	<1.56	<1.56	15.74 (12.5–25)
**6**	>50	2.45 (1.56–3.12)	39.68 (25–50)	19.84 (12.5–25)	19.84 (12.5–25)	4.95 (3.12–6.25)	2.47 (1.52–3.12)	<1.56	<1.56	31.49 (25–50)
**7**	>50	2.45 (1.56–3.12)	>50	15.75 (12.5–25)	19.84 (12.5–25)	2.47 (1.56–3.12)	4.95 (3.12–6.25)	2.47 (1.56–3.12)	31.49 (25–50)	39.68 (25–50)
**8**	>50	2.45 (1.56–3.12)	>50	39.68 (25–50)	19.84 (12.5–25)	31.49 (25–50)	2.47 (1.52–3.12)	<1.56	9.92 (6.25–12.5)	39.68 (25–50)
**9**	>50	>50	>50	9.92 (6.25–12.5)	39.68 (25–50)	>50	>50	<1.56	<1.56	4.95 (3.12–6.25)
GRI	2.52 (2–4)	0.198 (0.125–0.25)	3.175 (2–4)	1.26 (1–2)	3.174 (2–4)	>8	>8	>8	>8	1.587 (1–2)

*MICs are expressed as the geometric mean of three independent replicates. MIC range concentrations are reported within brackets. Tested strains are: Tmen: *Trichophyton mentagrophytes* CCF 4823, Tto: *Trichophyton tonsurans* CCF 4834, Trub*: Trichophyton rubrum* CCF 4879, Trub: *Trichophyton rubrum* CCF 4933, Tmen: *Trichophyton mentagrophytes* CCF 5930, Aqu: *Arthroderma quadrifidum* CCF 5792, Acro: *Arthroderma crocatum* CCF 5300, Acu: *Arthroderma curreyi* CCF 5207, Ains: *Arthroderma insigulare* CCF 5417, and Agyp: *Arthroderma gypseum* CCF 6261. Griseofulvin (GRI) was used as a reference control in these assays.

*T. mentagrophytes* (CCF 4823) and *T. rubrum* (CCF 4879) were found to be not susceptible to the TSC library, with the exception of compounds **4** and **6** on the first and second strains, respectively. Contrary, compound **5**, characterised by an indole ring, exerts the most potent activity against the tested panel and phenylsulfone **2** shows a good profile versus *Arthroderma* species, highlighting a selective behaviour.

### 3.2. Target prediction

The interesting biological profile of this class of compounds prompted us to investigate its mechanism of action and molecular targets. Thus, we performed a prediction analysis with the online Polypharmacology Browser PPB2^65^, highlighting, besides MAO-A and MAO-B, numerous putative targets, such as CAs.

### 3.3. Ca inhibition activity

Our large expertise in the characterisation and study of CAs also in the medicinal chemistry field, supported by the target prediction results and the strong chelating capability of TSCs, led us to investigate the interaction of our compounds library with CA enzymes and their zinc cofactor. Moreover, very recently, a docking study on a TSC derivative in the binding site of a βCA from a *Candida* species revealed that the zinc-binding ability of TSC moiety is minor than that of the sulfamoyl group present in the compound, ruling the pose, even if a relevant hydrogen-bonding pattern was established by the TSC nitrogen atoms with the enzyme^66^.

**Figure 2.**
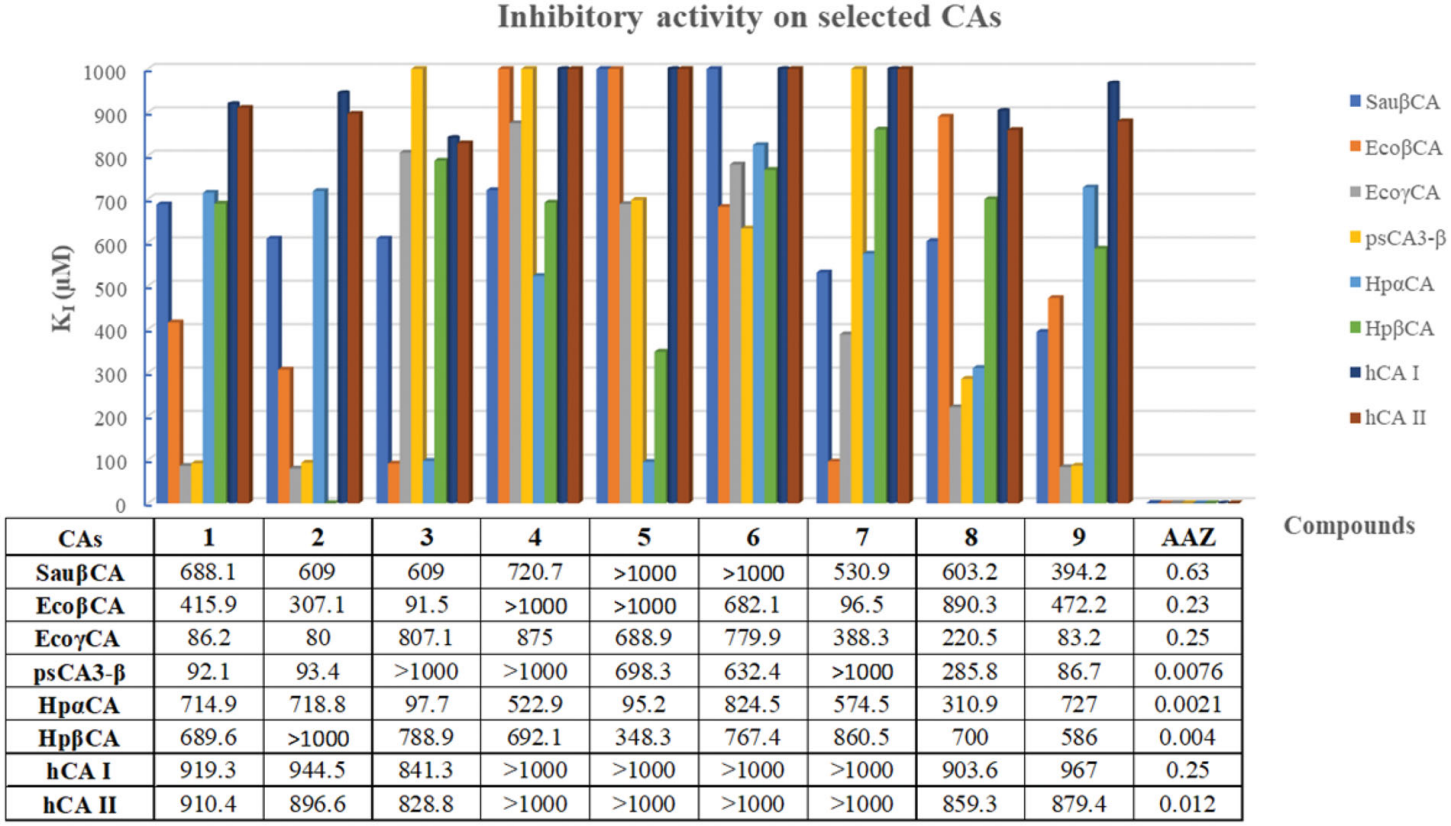
Inhibition data presented as histogram and *K*_I_ values of representative bacterial CAs and human isoforms (I and II) for TSCs **1–9** by the stopped-flow CO_2_ hydrase assay.

Hence, we assessed the inhibitory activity of compounds **1**–**9** on a panel of 6 bacterial CAs cloned and purified from *S. aureus* (SauβCA), *E. coli* (EcoβCA and EcoγCA), *P. aeruginosa* (psCA3-β), and *H. pylori* (HpαCA and HpβCA) and the human (h) CA I and II isoforms by means of the stopped-flow technique applied to the CO_2_ hydrase assay^67^. The inhibition data, compared to those of the standard sulphonamide inhibitor acetazolamide (AAZ), are reported in Figure 2.

Differently from the pan-isoform CA inhibitor AAZ, absent (*K*_I_ > 1000 μM) or very low (*K*_I_ > 800 μM) inhibitory activity was detected against hCAs I and II for TSCs **4**–**7** and **1**–**3**, **5**, **9**, respectively, highlighting a promising selectivity towards bacteria for this series with respect to the nanomolar pan-inhibitor AAZ. Compounds **1**, **2**, and **9** resulted to be selective on EcoγCA and psCA3-β with a selectivity index (*K*_I_ hCA I/*K*_I_ bacterial CA) of approximately 10 and this activity profile is reflected also in antibacterial susceptibility in *E. coli* ATCC 10536 for phenyl derivatives **1** and **2**.

TSCs **3** and **7** inhibit EcoβCA with good *K*_I_ values, while derivative **5** shows an interesting inhibitory activity on CAs from *H. pylori*. However, although the promising affinity and selectivity towards specific bacterial CAs, the antibacterial activity does not seem to be highly correlated to a unique CA-inhibition mode of action, even if it surely contributes to the biological profile of such compounds.

### 3.4. Absorption prediction

Finally, additional value to TSCs **1**–**9** was given by the prediction that they could cross the BBB and be absorbed by skin, as assessed through the BBB Predictor^68^ and SwissADME^69^ tools, respectively. These calculations could allow a further investigation for central nervous system (CNS) infections and confirm the applicability in infection-related skin diseases.

## 4. Conclusions

In the alarming frame of the COVID-19 pandemic, secondary infections and AMR are becoming a serious concern for Public Health. Several research programmes are now focussing on the development of new antimicrobial agents or the exploration of the antimicrobial properties of *in-house* libraries to propose new therapeutic strategies.

Encouraged by the widely reported multifaceted pharmacology of the TSC chemical class, we investigated the antimicrobial activity of an *in-house* small library of TSCs previously developed as anti-MAO agents. Thus, selected derivatives (**1**–**9**) were tested on a wide panel of infective bacterial and fungal strains. In particular, biological evaluations were performed on representative Gram-positive and Gram-negative bacterial strains, including environmental isolates, fungi belonging to *Candida* species, and dermatophytes, such as *Trichophyton* and *Arthroderma* species.

As regards the antibacterial profiles, several derivatives resulted to be very active against *E. coli* strains from the ATCC library (MICs range 2.45–9.92 µg/mL) and one environmental isolate (MICs range 7.87–9.92 µg/mL). Unfortunately, the good antibacterial profile is not maintained on a second isolate (MICs >50 µg/mL), suggesting one or more specific molecular targets but also the existence of at least one already developed resistant phenotype.

Although all the compounds were found inactive against *Candida* spp. at 50 µg/mL, interesting data were collected from the antidermatophytal susceptibility evaluation. In fact, several compounds exhibited potent activity against both *Trichophyton* and *Arthroderma* strains and the prediction of a good skin permeation could suggest their applicability in skin infectious diseases.

The investigation of the molecular mode of action of TSC compounds and our expertise in CAs drove us to assess the inhibitory activity of such derivatives towards selected CAs from bacteria. TSCs **1**, **2**, and **9** were found highly selective in inhibiting specific CA isoforms from *E. coli* and *P. aeruginosa*, whereas compounds **3** and **7** were found more potent against CAs of *H. pylori.* However, despite the interesting affinity and (human/bacterial) selectivity towards distinct CAs, their inhibition is expected to be one of several molecular targets, as suggested by the higher micromolar values of *K*_I_s.
